# The Gen-Equip Project: evaluation and impact of genetics e-learning resources for primary care in six European languages

**DOI:** 10.1038/s41436-018-0132-3

**Published:** 2018-07-27

**Authors:** Leigh Jackson, Anita O’Connor, Milena Paneque, Vaclava Curtisova, Peter W. Lunt, Radka Kremlíková Pourova, Milan Macek, Vigdis Stefansdottir, Daniela Turchetti, Mariana Campos, Lidewij Henneman, Lea Godino, Heather Skirton, Martina C. Cornel

**Affiliations:** 1 Faculty of Health and Human Sciences, Plymouth University, Plymouth, UK; 2 University of Exeter Medical School, RILD building, Royal Devon and Exeter Hospital, Exeter, United Kingdom; 3 i3S Instituto de Investigação e Inovação em Saúde, Institute for Molecular and Cell Biology (IBMC)—Centre for Predictive and Preventive Genetics (CGPP), Universidade do Porto, Porto, Portugal; 4 Dept. of Biology and Medical Genetics, Second Faculty of Medicine, Charles University and University Hospital Motol, Prague, Czech Republic; 5 Palacky University—University Hospital Olomouc, Olomouc, Czech Republic; 6 Landspitali National University Hospital, Reykjavik, Iceland; 7 Department of Medical and Surgical Sciences, Unit of Medical Genetics, University of Bologna, Bologna, Italy; 8 Genetic Alliance UK, London, United Kingdom; 9 Department of Clinical Genetics, Section Community Genetics and Amsterdam Public Health research institute, Amsterdam UMC location VUmc, Amsterdam, The Netherlands; 10 UO Oncologia, Policlinico Sant’Orsola Malpighi, Bologna, Italy

**Keywords:** primary care, online education, genetics, professional education, mixed methods

## Abstract

**Purpose:**

Genetic advances mean patients at risk of genetic conditions can be helped through testing, clinical screening, and preventive treatment, but they must first be identified to benefit. Ensuring quality of genetic care for patients requires genetic expertise in all health services, including primary care. To address an educational shortfall, a series of e-learning resources was developed in six languages to equip primary care professionals with genetic skills relevant for practice. The purpose of the study was to evaluate these resources using Kirkpatrick’s framework for educational outcomes.

**Methods:**

Mixed methods (qualitative and quantitative) were used over four phases of the study.

**Results:**

A high level of satisfaction with the resources was reported. Knowledge and skills improved significantly after using the education material. Participants reported changes in confidence and practice behavior, including family history taking, seeking advice from specialists and referring patients. The resources helped users to learn how to explain genetics. Many visited the resources repeatedly and some used them to educate colleagues or students.

**Conclusion:**

Gen-Equip modules are effective in improving genetic knowledge, skills, and attitudes for primary care professionals. They provide both continuing professional development and just-in-time learning for a potentially large global audience at a practical level.

## Introduction

Provision of appropriate health care for citizens is an important issue in every national context. While in the past, care of patients with genetic conditions was regarded as the province of genetics specialists, genetics and genomics are becoming increasingly embedded into health care in different settings. These include primary care,^1^ oncology,^2,3^ cardiology,^4^ and diabetes^5^ care. Approximately 30 million individuals in both Europe^6^ and the United States^7^ are affected by a rare disease and in 80.0% of those, genetic causes are a significant factor. Ensuring quality of care for these patients requires expertise at all levels of the health services, including primary care. In fact, conditions that can be detected in primary care settings are not rare. Familial hypercholesterolemia (FH) affects between 1/200 and 1/500 individuals in the general population,^8,9^ and this condition alone will be a risk to significant numbers of patients in every general practice. Easley et al.^10^ recently surveyed primary care and cancer physicians in Canada and reported that primary care practitioners required more training in cancer care, including with respect to the genetic testing relevant to treatment and management.

However, while the need is clear, there is also evidence that patients at risk of genetic disease may not be recognized by primary care professionals, resulting in delays in diagnosis and reduction in benefits from preventive or therapeutic interventions. For example, it is estimated that up to 85.0% of individuals with FH in the United Kingdom remain undiagnosed, and are being overlooked in primary care.^8,11^

A lack of knowledge and confidence in dealing with patients at risk of a genetic condition is reported in several recent studies in primary care.^1,12,13^

**Fig. 1:**
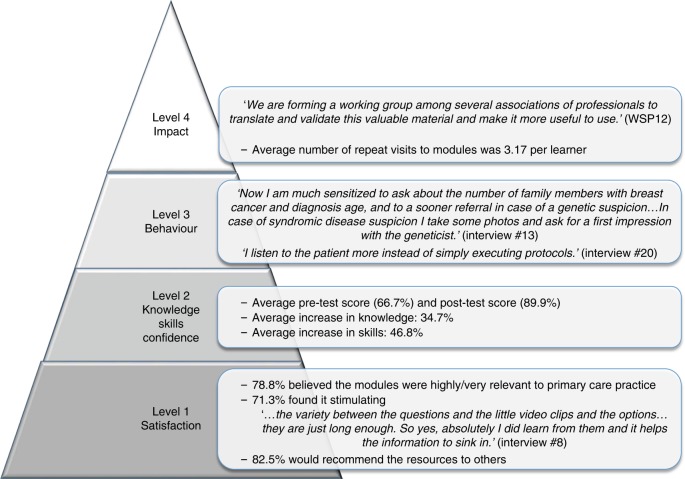
**Results of the evaluation of the Gen-Equip modules in relation to Kirkpatrick’s Evaluation Framework for Educational Outcomes.**^15,23^ Level 1: The degree to which participants find the training favorable, engaging and relevant to their jobs (Satisfaction). Level 2: The degree to which participants acquire the intended knowledge, skills, attitude, confidence, and commitment based on their participation in the training (Knowledge, Skills & Confidence). Level 3: The degree to which participants apply what they learned during training when they are back on the job (Behavior). Level 4: The degree to which targeted outcomes occur as a result of the training (Impact).^23^ *WSP*, workshop participant.

To achieve sustainable and innovative health care, service provision needs to be managed in a way that maximizes the impact of both time and resources^14^ and this is partly achieved by providing high quality training. The well-established Kirkpatrick’s model to assess effectiveness of training for health professionals includes the following components: satisfaction, and changes in knowledge, skills, and behavior (Fig. 1).^15^ The higher levels are relevant to assess impact on patient care. In a systematic review on genetics education for primary care,^16^ improvements in knowledge and confidence were reported in five and six studies respectively, but there was little evidence of changes in practice, which are more difficult to assess, and relied on self-assessment. Only in one study^17^ were changes in knowledge followed longitudinally.

To address the obvious shortfall in genetics education for primary care professionals, the Gen-Equip project (www.primarycaregenetics.org) developed accessible online training and resources to support primary care practitioners to develop and utilize their skills and knowledge of genetics and genomics for patient benefit. All aspects of project development are reported elsewhere.^18^ Primary care practitioners were defined as health professionals from medicine, nursing, midwifery, or allied health professions working in a primary care or community setting. This project involved partners from six European countries (Czech Republic, Iceland, Italy, Netherlands, Portugal and United Kingdom). The training and resources were made available in six languages and all were freely available online for use by practitioners from any discipline. The components included nine online modules (Table 1) that were based on cases that would typically be seen in primary care and were highly interactive, including videos, quizzes, and web links to external resources.^18^ Webinars (of 20–30 min) on six topics relevant to primary care, such as family history taking, were recorded and could be watched at any time from the website. In addition, resources were prepared for use in daily practice, such as referral guidelines, family history taking tools, and short videos on how to explain genetic concepts to patients.

**Table 1:** List of online modules

Familial Breast and Ovarian Cancer
Familial Colon Cancer
Inherited Cardiac Conditions
Familial Hypercholesterolemia
A child with a genetic condition
Pregnancy 1.—Assessing risk of a genetic condition in the fetus where there is a family history
Pregnancy 2.—Assessing risk of a genetic condition in the fetus where there is no family history
Pregnancy 3.—Impact of medication or maternal medical conditions on the fetus
Pregnancy 4.—Chromosomal conditions in the fetus

### Aim of the study

The aim of this study was to assess the effectiveness of the Gen-Equip case-based online interactive modules.

The objectives were to assess learners'

- Satisfaction with the training
- Changes in knowledge and skills
- Changes in perceived confidence in providing genetic health care
- Changes in self-reported clinical practice behavior
- Changes affecting the wider profession or health-care community

## Materials and methods

Evaluation of the educational materials was integral to the Gen-Equip project. The project team assessed changes that could affect care of patients in four phases, using different methods. The use of mixed methods is common in health services research^19–21^ where the impact of any intervention is likely to be both complex and where some impact is difficult to measure. Both quantitative and qualitative methods were used to collect and analyze data, improving the trustworthiness of the findings through triangulation.^22^

Learners who wished to take one or more of the free, online modules were directed to Open Digital Learning Environment hosted by Plymouth University. All those wishing to access a module were asked to register, using their first and last name and email address. They were then able to access the modules as many times as they wished. The website tracked users’ activity, which resources they accessed, and how often. Fig. 1 shows the relationship between Kirkpatrick’s model for evaluating educational interventions and the results.^15^

### Phase 1. Differences in pre- and postmodule knowledge and skills

Study design: Pre- and postintervention test. All learners who registered for any of the nine e-learning modules were invited to take a pre- and postintervention test of knowledge and skills (Kirkpatrick level 2).^23^ Those who achieved at least 80.0% on the postmodule test were awarded a certificate of completion.

Participants: All health professionals who undertook a module were eligible to take part.

Data collection: The aim was to obtain >350 sets of pre- and postmodule test scores for the analysis. Only scores from individuals who completed both pre- and postmodule tests before October 2017 have been included in the present analysis. The premodule scores were used to assess baseline knowledge and skills, and differences in pre- and postmodule scores to assess the extent to which the module was effective in improving knowledge and skills in the short term. A restriction on the premodule test was set to enable learners to do that once only. Prior to this restriction, it was observed that learners were completing the premodule test multiple times. There was no apparent score increase when the test was repeated and thus it was decided there was no need to adjust the statistical testing. The overall change in knowledge and skills across the entire set of modules was analyzed, as well as for the individual modules. Modules were designed by a multidisciplinary group of patient representatives, and primary care, genetics, and education specialists. The topics and questions were informed by solicited opinions from primary care on the Gen-Equip website, existing curricula and competencies set by professional organizations in primary care. Quality was assured by obtaining accreditation by the Royal College of General Practitioners (RCGP) and European Accreditation Network (EAN). Examples of knowledge questions and skills questions are given in Supplementary Table S1.

### Phase 2. Postmodule feedback survey

Study design: Cross-sectional survey.

Participants: All learners who registered for a module were asked to provide feedback via an online survey.

Data collection: The survey was set up using Survey Monkey software and comprised 22 questions. The survey tool was developed by the project partners based on the literature review and a stakeholder workshop in November 2014. The survey questions focused on demographic data, whether the educational needs of primary care practitioners were met (Kirkpatrick level 1 [ref.^23^]), whether they found the online delivery method acceptable, and had been able to apply the knowledge to their clinical practice (Kirkpatrick level 3 [ref.^23^]). The majority of questions were multiple choice but there were several free-text questions to enable participants to give their own views on changes to their practice, what they liked most about the modules, and suggestions for improvement.

### Phase 3. Feedback from workshop attendees

Study design: Cross-sectional surveys.

Participants: In May 2017, we held a one-day “multiplier event,” a workshop titled “Sharing Best Practice: Enabling Primary Care Health Professionals to Deal with Genetics” for 61 participants from 14 countries. All those who attended were professionals with a strong interest in promoting genetics education for primary care. The background of attendees included primary care professionals, genetics health specialists, and health professional educators.

Data collection: Participants were asked to complete a paper survey at the end of the workshop (*N* = 44, response rate 72.1%) and an online survey (using Survey Monkey) 4 months later, indicating how they had used the resources in clinical practice and in teaching (*N* = 35, response rate 57.4%).

### Phase 4. Qualitative study

Study design: Interviews were used to conduct this qualitative study.

Participants: Learners who completed a module successfully were contacted by email 2–3 months after completion of the module and asked whether they would be willing to engage in a telephone or Skype interview. A participant information sheet was included with the invitation. Participants who were willing to be interviewed then contacted the researcher. We aimed to recruit a maximum variation sample, with participants from all partner countries and with a range of professions, ages, and years of experience in health-care practice. Data were collected until saturation was reached after 21 interviews.

Data collection: We used a semistructured interview schedule to explore the following topics: learners’ experience of the module, perceived increase in knowledge and skills, usefulness for daily practice, and suggestions for improvements. Nineteen interviews were digitally recorded with the consent of the participant: two respondents sent their replies by email.

### Data analyses

Scores were collected, entered into statistical analysis software SPSS version 24 and subjected to analysis. Descriptive statistical tests (means and standard deviations) were used to analyze demographic data and scores of knowledge, skills and satisfaction. For the pre- and postintervention test (phase 1) paired samples *t* tests were used to assess differences in scores. Thematic analysis^24^ was used to analyze the free-text responses in the surveys. The interview data were transcribed verbatim, anonymized, and analyzed thematically by hand.^24^ The process of data collection and analysis ran concurrently. Each statement was coded independently by three researchers, who met to discuss the analysis and group the data into categories and finally themes.

### Ethics

?tlsb .015w?>Ethical approval for the study was obtained from the University of Plymouth Faculty of Health and Human Sciences Research Ethics Committee. All data were collected, used, and reported anonymously. All learners were informed as they entered the module pages that the test scores would be used anonymously to assess the learning materials and that if they proceeded to take the tests, then consent would be presumed. Survey participants and interviewees were provided with information about the study prior to agreeing to take part and were made aware that they could withdraw at any time. Consent to record interviews was obtained. Participant details were altered to ensure anonymity.

## Results

To the end of October 2017, there were 7349 unique visits to the website. Visitors originated from 110 countries and all populated continents: 5066 (68.9%) were from Europe. A total of 1330 visitors used the link from the www.primarycaregenetics.org website to the Gen-Equip online interactive modules. Visitors to the website were directed to other types of resources and 1989 used these links.

The demographic data of the respondents are presented in Table 2. Demographic data are not available from phase 1, as the scores were completely anonymized.

**Table 2:** Demographic characteristics of respondents

		Phase 2 online user survey *N*=81		Phase 3 workshop participants who completed the online survey *N*=35		Phase 4 participants interviewed *N*=21	
Category	Options	*N*	%	*N*	%	*N*	%
Gender	Female	72	88.9	28	80.0	19	90.5
	Male	8	9.88	7	20.0	2	9.52
	No answer	1	1.23	0		0	
Age (years)	20–29	14	17.3	9	25.7	3	14.3
	30–39	20	24.7	14	40.0	10	47.6
	40–49	16	19.8	3	8.57	4	19.0
	50–59	22	27.2	7	20.0	2	9.52
	60 years and over	9	11.1	1	2.86	1	4.76
	No answer			1	2.86	1	4.76
Main professional qualification	Medicine	25	30.9	19	54.3	12	57.1
	Nursing	34	42.0	3	8.57	7	33.3
	Midwifery	6	7.41	1	2.86	0	
	Other	16	19.8	12	34.3	1	4.76
	No answer					1	4.76
Place of work^a^	A primary care clinic	19	23.5	8	22.9	8	38.1
	A community health center	9	11.1	2	5.71	6	28.6
	A district or community hospital	7	8.64	5	14.3	1	4.76
	An acute hospital	29	35.8	3	8.57	6	28.6
	An educational organization	6	7.41	9	25.7	3	14.3
	Other	10	12.3	8	22.9	2	9.52
	No answer	1	1.23				
Years as health professional	0–5	26	32.1	NA		5	23.8
	6–10	8	9.88			6	28.6
	11–15	5	6.17			1	4.76
	16–20	11	13.6			4	19.0
	21–25	5	6.17			1	4.76
	>25	26	32.1			3	14.3
	No answer					1	4.76
Country	Czech Republic	6	7.41	2	5.71	1	4.76
	Iceland	2	2.47	1	2.86	0	
	Italy	2	2.47	1	2.86	3	14.3
	Netherlands	2	2.47	4	11.4	2	9.52
	Portugal	8	9.88	9	25.7	5	23.8
	United Kingdom	36	44.4	8	22.9	8	38.1
	Other	25	30.9	9	25.7	2	9.52
	No answer			1	2.86		

*NA*, not assessed

^a^Numbers may exceed total participants as individuals may work in more than in one setting

We present the results of the study according to Kirkpatrick’s model^15^ (Fig. 1). Against the quotes, WSP denotes “workshop participant,” while qualitative interviewees are marked as “interview.”

### Satisfaction with the resources

Satisfaction with the resources was assessed during phases 2–4.

Eighty learners from 14 countries responded to a survey on the learning resources. Of the respondents, 73.8% reported the module was highly or very relevant to their own clinical practice, while 78.8% believed it was highly or very relevant to primary care practice generally and 76.3% reported the resources matched well to the primary care curriculum. That the content was extremely or very clear was reported by 83.8%, and that the resources were easy to navigate by 78.8%, while 87.5% reported that the resources were presented with an appropriate level of detail. Respondents were asked how they felt after doing the module; 53.8% found it enjoyable, 71.3% found it stimulating, 1.30% found it boring, 1.30% not enjoyable, and 1.30% were frustrated (more than one response could be given). When asked if they would recommend the resources to a colleague, 82.5% said they would do so.

These results were confirmed by workshop attendees and interviewed learners, who reported that the Gen-Equip resources were useful, interactive, easy to access/use, and applicable to practice. The simple wording made the resources accessible to nongenetic health professionals and helped the user to learn how to explain genetics to students, patients, and their families. Respondents reported that they returned to the modules repeatedly in practice when they wanted to confirm whether a patient was in a high risk group. The possibility of accessing the resources as often as needed and the varied presentation and interactivity were also appreciated.

Interviewees also reported that they had recommended the modules to others. However, some queried whether lack of time might prevent their use by general practictioners (GPs), with feedback that some of the modules were quite long.

### Changes in knowledge and skills

Changes in knowledge and skills were assessed via the pre- and postmodule tests. The results for the English modules until mid-October 2017 are presented in Table 3.

**Table 3:** Results: pre- and postmodule scores

	Overall results					Scores related to questions on knowledge					Scores related to questions on skills				
Module	*N*	Premodule score (%)	Postmodule score (%)	% change	Paired *t* test significance	*N* ^a^	Premodule score (%)	Postmodule score (%)	% change	Paired *t* test significance	*N* ^a^	Premodule score (%)	Postmodule score (%)	% change	Paired *t* test significance
Familial Breast and Ovarian Cancer	120	68.0	90.6	33.3	<0.001	600	63.3	88	38.9	<0.001	600	72.6	93.1	28.3	<0.001
Familial Colon Cancer	104	56.7	87.0	53.5	<0.001	832	56.7	88.8	56.7	<0.001	208	57.6	79.8	40.7	<0.001
Pregnancy 1	44	76.6	93.5	22.0	<0.001	132	74.3	92.7	24.8	<0.001	176	78.4	94.0	19.9	<0.001
Pregnancy 2	41	60.5	88.4	46.1	<0.001	205	67.2	86.6	28.7	<0.001	41	27.6	97.5	264	<0.001
Pregnancy 3	27	57.9	91.4	57.9	<0.001	162	52.3	90.6	73.2	<0.001	27	91.4	96.3	5.40	0.255
Pregnancy 4	32	80.5	93.5	16.1	0.001	96	77.1	91.3	18.5	<0.001	96	84.3	95.6	13.9	<0.001
Inherited Cardiac Conditions	34	75.3	90.5	20.2	0.003	68	74.3	94.1	26.7	<0.001	204	76.5	89.3	18.0	<0.001
Familial Hypercholesterolemia	17	68.6	88.0	28.3	<0.001	102	64.9	86.3	32.8	<0.001	102	71.0	89.2	25.5	<0.001
Child with a Genetic Condition	4	81.0	87.9	8.60	0.526	20	80.8	90.0	11.3	0.085	20	81.0	85.8	5.80	0.418
**Total**	423	66.7	89.9	23.2	<0.001										

^a^Per question

The number of people taking each module varied from 4 to 120 and the numbers correlated to how long each module was live on the website. Combining data from all modules (*N* = 423) the average pretest score was 66.7% and the average posttest score was 89.9% with a paired *t* test significance of *p* < 0.001. In every module a statistically significant increase in scores was observed apart from the module on a child with a genetic condition, where the sample size was too small, due to being the last module to go live and only having a few days available to learners. For this reason we have excluded this module from further discussion here. The modules with the lowest premodule scores were those covering familial colorectal cancer and the pregnancy module 3, on risk to the health of the fetus due to maternal conditions or medication. These modules also showed the largest percentage increase in score posttest (53.5 and 57.9% respectively).

The questions on the tests were split into two categories, those that were inquiring about clinical skills and those that related purely to knowledge. Average increase in skills and knowledge was 46.8% and 34.7%, respectively.

When considering the skills-based questions, all modules generated a significant increase in the posttest scores apart from, rather unexpectedly, the pregnancy module 3. Despite the overall low pretest score in this module, the skills questions were answered extremely well, demonstrating that the issue with this topic is a severe knowledge deficit.

When considering the knowledge-based questions, every module resulted in a significant increase in posttest scores: greatest differences were seen in the colorectal cancer and pregnancy 3 modules (56.7% and 73.2% respectively). Across all categories (overall, skills, and knowledge), the average mark posttest was above the pass mark of 80.0% in all modules. This pass mark was set in accordance with previous observations of the need for medical education to be challenging, as well as reflecting approximately the pass marks used by professional clinical bodies for their exams. In the pretests, only the pregnancy 4 module had an average mark that would pass the threshold. The premodule quizzes had pass rates of between 9.10 and 64.9% (for full list see Supplementary Table S2) by module-naïve learners. Whilst each module was equally academically difficult to pass, these differences may reflect the difficulties learners have with certain topics or conversely that they may have favored taking modules they were already familiar or interested in.

The number of learners completing more than one module (keeners) was 387/502 (77.1%) with 43 (8.57%) doing four or more modules and two learners completing all nine. Due to our desire to collect minimal personal information about individual learners we are unable to characterize further any differences between keeners and single-module learners.

Learners who completed the postmodule test and achieved a score ≥80.0% were awarded a certificate to demonstrate their continuing professional development. This was optional and not all learners completed the test. By October 2017 >600 completion certificates had been awarded.

Despite having already passed the postmodule quiz, learners revisited the modules at later dates. The average number of visits to each module per learner was between 2.84 and 3.82 (see Supplementary Table S3 for breakdown). For some of the qualitative study interviewees, the modules complemented and refreshed what they had already learned. However, many reported that their knowledge of genetic issues had changed. Examples given were an increase in their knowledge on and awareness of “red flags” that should raise suspicion of a genetic condition, referral criteria, understanding of genetic aspects of disease and inheritance patterns, and knowing what to ask when taking a family history.

According to some interviewees, the modules also increased awareness of patients’ perspectives and preferences:

“I am much more aware when I am talking to patients about genetic testing, about the wider implications […] understanding the emotional impact it has on patients.” (interview 10)

### Changes in confidence

From the interviews, it became clear that following the modules increased learners’ confidence in talking about genetic results and genetic testing with families and when taking a family history:

“[patients] come in and they are concerned about cancer, breast cancer or ovarian cancer, there is a strong family history, and do I really need to refer them?……because you have more knowledge and you think about what questions to ask and you feel more confident when you make that assessment, you feel more up-to-date.“ (interview 9)

Others felt more confident in teaching, integrating genetics in their own education for students:

“Not only for me, in my practice, but more for what I am teaching the students….” (interview 6)

### Changes in practice behavior

When asked how the Gen-Equip modules had influenced their practice and teaching, many cited that they had made them more likely to “be suspicious” of a genetic condition where this was possible, to ask more questions about the family history, to seek advice from genetics colleagues, and to refer to specialist services (Fig. 1). Others paid more attention to the patient’s perspective:

“I do leave options open more and I talk to people about why they might not want to be tested, [stating that] they don’t have to do this but this is an option for you…it has changed my practice with each patient.” (interview 3)

### Changes beyond the individual practitioner (impact)

Four months after the Gen-Equip workshop, attendees reported using the Gen-Equip resources in three main ways outside their own practice. First, they had influenced other practitioners to use the resources:

“As member of the [country] Network of Public Health Genomics I will disseminate the Gen-Equip resources among other members of the Network.”(WSP21)

Second, they had utilized the resources for educational purpose:

“I teach…GPs on genetics and this course has changed my focus more towards equipping GPs with more clinically relevant genetics information.” (WSP11)

Third, they had used Gen-Equip as a vehicle to form stronger links between primary care and genetics in their own countries, by writing papers, organizing workshops, and/or forming new working groups (Fig. 1):

“I have written an article to […] Medical Journal about the educational needs in medical genetics in family practice.” (WSP4)

## Discussion

In both designing the evaluation and our analyses, we aligned the process to the model of assessment of educational interventions developed by Kirkpatrick^23^ and others^15^ (Fig. 1).

The Gen-Equip materials demonstrated to be effective in increasing knowledge and skills, and helped to implement changes in practice, such as systematic family history taking and helping patients making their own informed decision. Other authors^25,26,27^ have emphasized that primary care professionals require materials they can access “just in time,” at the point of patient contact. Although a proportion of learners wished to be issued with a certificate to demonstrate their continuing professional development, many used Gen-Equip resources as an ongoing source of information, and went back to the materials repeatedly. The observation that over 75% of learners were “keeners” and nearly 10% completed more than four modules speaks to the practicality of the tool for primary care clinicians and the ease of integrating its use into their practice.

The data on acceptability of the resources confirms the findings of other authors^28^ that health professionals find e-learning an acceptable method of improving their knowledge. We used some principles of online learning of Sheringham et al.,^2^ setting high expectations (by using a high pass mark), ensuring learning materials encouraged active participation (using interactive features), giving feedback throughout the modules regarding correct or incorrect choices, and addressing the diverse range of learners by including optional material. As well as being effective, as demonstrated by increasing test scores, participants confirmed that these strategies were helpful and stimulating.

Health-care professionals tend to lack knowledge of genetics that is relevant for daily practice.^29^ In a systematic review on the impact of e-learning resources on health professional behavior and on patient outcomes, Sinclair et al.^30^ concluded that generally e-learning could be as effective as traditional learning modes but that this depended on the skills being taught. While live training may be more effective in improving consultation skills,^31^ e-learning enables a greater number of practitioners to access the courses^32^ and potentially benefit a larger audience.

Sinclair et al.^30^ did not identify any studies where patient outcomes had been assessed and only one study reporting longitudinal changes in health professional behavior.^33^ We were not able to assess direct impact on patients through this evaluation, however there was self-reported evidence from practitioners that their behavior with respect to detecting patients at risk and seeking further advice from specialists had been affected, as long as 6 months after using the Gen-Equip resources.

### Limitations of the study

Our workshop participants (phase 3) were a biased group as compared with average primary care workers, as those had a demonstrable interest in genetics. Although participants in phases 1, 2, and 4 were derived from a more general population of health professionals, they may also have had a preexisting interest in genetics.

There are, of course, limitations to delivery of clinical education via e-learning, but we noted that even attitudes appeared to have been affected by the use of the resources, for example, changes in understanding that patients may view genetic testing as negative as well as positive, and that testing should be seen by the patient as an option.

With regard to changes in clinical and educational practice, we have obtained data for up to 6 months postmodule, and we are unable to show whether the changes would persist beyond that time. In addition, many changes in practice behavior are self-reported and qualitative. For some of the self-reported educational activities there was independent evidence in the corresponding increase in module usage. To evaluate patient health impact at the highest Kirkpatrick^23^ level, further empirical work is needed, for example to assess changes in detection of patients at genetic risk and rates of relevant referral to specialist services. This was outside the scope of the current project.

### Conclusion

We have demonstrated that online training is effective in changing knowledge, skills, and attitudes of health professionals. However, one aspect not measured by Kirkpatrick’s model was the impact of raising awareness of genetic health issues that was achieved through the project, which can be built on with further work.

The universally significant increase in knowledge demonstrates the comprehensive nature of the intervention and the increases obtained can potentially translate to substantial real-world clinical impact if this extra knowledge is operationalized.

As genetics is gaining importance in many fields of medicine, online training may help to reach a relatively large group of learners for continuing professional development.

## Supplementary information

Supplementary Table 1

Supplementary Table 2

Supplementary Table 3
