# Correction: The Gen-Equip Project: evaluation and impact of genetics e-learning resources for primary care in six European languages

**DOI:** 10.1038/s41436-018-0279-y

**Published:** 2018-08-23

**Authors:** Leigh Jackson, Anita O’Connor, Milena Paneque, Vaclava Curtisova, Peter W. Lunt, Radka Kremlíková Pourova, Milan MacekJr, Vigdis Stefansdottir, Daniela Turchetti, Mariana Campos, Lidewij Henneman, Lea Godino, Heather Skirton, Martina C. Cornel

**Affiliations:** 1 Faculty of Health and Human Sciences, Plymouth University, Plymouth, UK; 2 University of Exeter Medical School, RILD building, Royal Devon and Exeter Hospital, Exeter, United Kingdom; 3 i3S Instituto de Investigação e Inovação em Saúde, Institute for Molecular and Cell Biology (IBMC)—Centre for Predictive and Preventive Genetics (CGPP), Universidade do Porto, Porto, Portugal; 4 Dept. of Biology and Medical Genetics, Second Faculty of Medicine, Charles University and University Hospital Motol, Prague, Czech Republic; 5 Palacky University—University Hospital Olomouc, Olomouc, Czech Republic; 6 Landspitali National University Hospital, Reykjavik, Iceland; 7 Department of Medical and Surgical Sciences, Unit of Medical Genetics, University of Bologna, Bologna, Italy; 8 Genetic Alliance UK, London, United Kingdom; 9 Department of Clinical Genetics, Section Community Genetics and Amsterdam Public Health research institute, Amsterdam UMC location VUmc, Amsterdam, The Netherlands; 10 UO Oncologia, Policlinico Sant’Orsola Malpighi, Bologna, Italy

Correction to: Genetics in Medicine; 10.1038/s41436-018-0132-3 published online 27 July 2018

This Article was originally published under Nature Research’s License to Publish, but has now been made available under a [CC BY 4.0] license. The PDF and HTML versions of the Article have been modified accordingly.

