# Supplementary material for: The Gen-Equip Project: evaluation and impact of genetics e-learning resources for primary care in six European languages
Source: Genet Med. 2018 Jul 27;21(3):718–26. doi: 10.1038/s41436-018-0132-3 (PMC6752302; doi:10.1038/s41436-018-0132-3)
Supplement: Supplementary file 1 — Supplementary Table 1 [file 41436_2018_132_MOESM1_ESM.docx]

**Table 1 List of online modules**

| Familial Breast and Ovarian Cancer  Familial Colon Cancer  Inherited Cardiac Conditions  Familial Hypercholesterolaemia  A child with a genetic condition  Pregnancy 1. - Assessing risk of a genetic condition in the fetus where there is a family history  Pregnancy 2. - Assessing risk of a genetic condition in the fetus where there is no family history  Pregnancy 3. - Impact of medication or maternal medical conditions on the fetus  Pregnancy 4. - Chromosomal conditions in the fetus |
| --- |
