# Supplementary material for: The Gen-Equip Project: evaluation and impact of genetics e-learning resources for primary care in six European languages
Source: Genet Med. 2018 Jul 27;21(3):718–26. doi: 10.1038/s41436-018-0132-3 (PMC6752302; doi:10.1038/s41436-018-0132-3)
Supplement: Supplementary file 2 — Supplementary Table 2 [file 41436_2018_132_MOESM2_ESM.docx]

**Table 2 Demographic characteristics of respondents**

|  |  | **Phase 2**  **Online user survey**  **N=81** | | **Phase 3**  **Workshop participants who completed the online survey**  **N=35** | | **Phase 4**  **Participants interviewed**  **N=21** | |
| --- | --- | --- | --- | --- | --- | --- | --- |
| Category | Options | N | % | N | % | N | % |
| Gender | Female | 72 | 88.9 | 28 | 80.0 | 19 | 90.5 |
|  | Male | 8 | 9.88 | 7 | 20.0 | 2 | 9.52 |
|  | No answer | 1 | 1.23 | 0 |  | 0 |  |
|  |  |  |  |  |  |  |  |
| Age (years) | 20-29 | 14 | 17.3 | 9 | 25.7 | 3 | 14.3 |
|  | 30-39 | 20 | 24.7 | 14 | 40.0 | 10 | 47.6 |
|  | 40-49 | 16 | 19.8 | 3 | 8.57 | 4 | 19.0 |
|  | 50-59 | 22 | 27.2 | 7 | 20.0 | 2 | 9.52 |
|  | 60 years and over | 9 | 11.1 | 1 | 2.86 | 1 | 4.76 |
|  | No answer |  |  | 1 | 2.86 | 1 | 4.76 |
|  |  |  |  |  |  |  |  |
| Main professional qualification | Medicine | 25 | 30.9 | 19 | 54.3 | 12 | 57.1 |
|  | Nursing | 34 | 42.0 | 3 | 8.57 | 7 | 33.3 |
|  | Midwifery | 6 | 7.41 | 1 | 2.86 | 0 |  |
|  | Other | 16 | 19.8 | 12 | 34.3 | 1 | 4.76 |
|  | No answer |  |  |  |  | 1 | 4.76 |
|  |  |  |  |  |  |  |  |
| Place of work^a^ | A primary care clinic | 19 | 23.5 | 8 | 22.9 | 8 | 38.1 |
|  | A community health centre | 9 | 11.1 | 2 | 5.71 | 6 | 28.6 |
|  | A district or community hospital | 7 | 8.64 | 5 | 14.3 | 1 | 4.76 |
|  | An acute hospital | 29 | 35.8 | 3 | 8.57 | 6 | 28.6 |
|  | An educational organisation | 6 | 7.41 | 9 | 25.7 | 3 | 14.3 |
|  | Other | 10 | 12.3 | 8 | 22.9 | 2 | 9.52 |
|  | No answer | 1 | 1.23 |  |  |  |  |
|  |  |  |  |  |  |  |  |
| Years as health professional | 0-5 | 26 | 32.1 | NA |  | 5 | 23.8 |
|  | 6-10 | 8 | 9.88 |  |  | 6 | 28.6 |
|  | 11-15 | 5 | 6.17 |  |  | 1 | 4.76 |
|  | 16-20 | 11 | 13.6 |  |  | 4 | 19.0 |
|  | 21-25 | 5 | 6.17 |  |  | 1 | 4.76 |
|  | >25 | 26 | 32.1 |  |  | 3 | 14.3 |
|  | No answer |  |  |  |  | 1 | 4.76 |
|  |  |  |  |  |  |  |  |
| Country | Czech Republic | 6 | 7.41 | 2 | 5.71 | 1 | 4.76 |
|  | Iceland | 2 | 2.47 | 1 | 2.86 | 0 |  |
|  | Italy | 2 | 2.47 | 1 | 2.86 | 3 | 14.3 |
|  | Netherlands | 2 | 2.47 | 4 | 11.4 | 2 | 9.52 |
|  | Portugal | 8 | 9.88 | 9 | 25.7 | 5 | 23.8 |
|  | United Kingdom | 36 | 44.4 | 8 | 22.9 | 8 | 38.1 |
|  | Other | 25 | 30.9 | 9 | 25.7 | 2 | 9.52 |
|  | No answer |  |  | 1 | 2.86 |  |  |

^a^ Numbers may exceed total participants as individuals may work in more than in one setting.

NA: Not assessed.
