# Supplementary material for: The Gen-Equip Project: evaluation and impact of genetics e-learning resources for primary care in six European languages
Source: Genet Med. 2018 Jul 27;21(3):718–26. doi: 10.1038/s41436-018-0132-3 (PMC6752302; doi:10.1038/s41436-018-0132-3)
Supplement: Supplementary file 3 — Supplementary Table 3 [file 41436_2018_132_MOESM3_ESM.docx]

**Table 3 Results: pre- and post-module scores**

|  | **Overall results** | | | | | **Scores related to questions on knowledge** | | | | | **Scores related to questions on skills** | | | | |
| --- | --- | --- | --- | --- | --- | --- | --- | --- | --- | --- | --- | --- | --- | --- | --- |
| Module | N | Pre-module score (%) | Post-module score (%) | % change | Paired  t-test significance | N^a^ | Pre-module score (%) | Post-module score (%) | % change | Paired  t-test significance | N^a^ | Pre-module score (%) | Post-module score (%) | % change | Paired  t-test significance |
| Familial Breast and Ovarian Cancer | 120 | 68.0 | 90.6 | 33.3 | <0.001 | 600 | 63.3 | 88 | 38.9 | <0.001 | 600 | 72.6 | 93.1 | 28.3 | <0.001 |
| Familial Colon Cancer | 104 | 56.7 | 87.0 | 53.5 | <0.001 | 832 | 56.7 | 88.8 | 56.7 | <0.001 | 208 | 57.6 | 79.8 | 40.7 | <0.001 |
| Pregnancy 1 | 44 | 76.6 | 93.5 | 22.0 | <0.001 | 132 | 74.3 | 92.7 | 24.8 | <0.001 | 176 | 78.4 | 94.0 | 19.9 | <0.001 |
| Pregnancy 2 | 41 | 60.5 | 88.4 | 46.1 | <0.001 | 205 | 67.2 | 86.6 | 28.7 | <0.001 | 41 | 27.6 | 97.5 | 264 | <0.001 |
| Pregnancy 3 | 27 | 57.9 | 91.4 | 57.9 | <0.001 | 162 | 52.3 | 90.6 | 73.2 | <0.001 | 27 | 91.4 | 96.3 | 5.40 | 0.255 |
| Pregnancy 4 | 32 | 80.5 | 93.5 | 16.1 | 0.001 | 96 | 77.1 | 91.3 | 18.5 | <0.001 | 96 | 84.3 | 95.6 | 13.9 | <0.001 |
| Inherited Cardiac Conditions | 34 | 75.3 | 90.5 | 20.2 | 0.003 | 68 | 74.3 | 94.1 | 26.7 | <0.001 | 204 | 76.5 | 89.3 | 18.0 | <0.001 |
| Familial Hyper-cholesterolaemia | 17 | 68.6 | 88.0 | 28.3 | <0.001 | 102 | 64.9 | 86.3 | 32.8 | <0.001 | 102 | 71.0 | 89.2 | 25.5 | <0.001 |
| Child with a genetic condition | 4 | 81.0 | 87.9 | 8.60 | 0.526 | 20 | 80.8 | 90.0 | 11.3 | 0.085 | 20 | 81.0 | 85.8 | 5.80 | 0.418 |
| **Total** | 423 | 66.7 | 89.9 | 23.2 | <0.001 |  |  |  |  |  |  |  |  |  |  |

^a^Per question.
